# Re: Discharge NIHSS scores are predictive of poor 3-month outcomes in patients with acute ischemic stroke receiving intravenous thrombolysis

**DOI:** 10.1080/07853890.2026.2617750

**Published:** 2026-01-19

**Authors:** Yang Duan

**Affiliations:** The First Affiliated Hospital of Hebei North University, Zhangjiakou, Hebei Province, China

To the editor,

I read with great interest the study by Zhu et al. which provides a thoughtful and clinically meaningful evaluation of discharge NIHSS as a prognostic indicator in thrombolysis-treated acute ischemic stroke [1]. The authors demonstrate, with rigorous statistical analysis, that discharge NIHSS shows superior discriminative performance compared with baseline, 24-hour, and early change metrics in predicting 90-day functional outcomes. I believe this work addresses an important gap in post-thrombolysis prognostication. At the same time, several study-specific conceptual and analytical issues deserve further consideration.

First, I note that discharge NIHSS represents a post-treatment composite surrogate rather than a conventional baseline predictor. Unlike baseline or fixed-time NIHSS measures, discharge NIHSS inherently integrates treatment response, in-hospital complications, recovery dynamics, and clinical discharge decisions. Treating it as an independent prognostic variable alongside baseline and 24-hour NIHSS may introduce post-treatment conditioning and causal ambiguity. In my view, the observed superiority of discharge NIHSS likely reflects its role as a summary measure of early recovery rather than intrinsic predictive dominance. Explicitly positioning discharge NIHSS within a causal or mediation framework—linking baseline severity, early neurological change, and stabilized in-hospital outcomes—would strengthen mechanistic interpretation and avoid overextension of predictive claims [2].

Second, I believe that unmodeled discharge timing introduces systematic heterogeneity that may inflate discriminative performance. Because discharge NIHSS is inherently time-dependent, ignoring length of stay and discharge timing may advantage this metric over fixed-time assessments in ROC comparisons. Patients discharged earlier versus later with similar NIHSS scores may represent fundamentally different recovery trajectories and risk profiles. Accounting for discharge timing, performing landmark analyses at standardized post-onset intervals, or stratifying analyses by length-of-stay categories would improve fairness of comparison and enhance generalizability across health systems with diverse discharge practices [3].

Third, I suggest that the emphasis on an optimized cutoff value (NIHSS ≥4.5) warrants additional statistical and clinical contextualization. While this threshold is statistically optimal by Youden index, its non-integer nature limits direct clinical interpretability and may be sensitive to sampling variability. Reporting uncertainty around the cutoff, comparing adjacent integer thresholds, and supplementing ROC analysis with decision-curve analysis would better align statistical optimization with real-world clinical decision-making [4].

Finally, I propose that discharge NIHSS be viewed not only as a prognostic marker but also as a transition-point indicator linking acute care to recovery-oriented interventions. Patients identified at discharge with residual neurological deficits may benefit from interdisciplinary, nurse-led approaches informed by social-art interventions—such as music, visual art, or structured expressive activities—that support motivation, emotional regulation, and engagement in rehabilitation [5]. Integrating discharge NIHSS–based risk stratification with such human-centered nursing practices may extend prognostic models beyond outcome prediction toward active optimization of recovery trajectories.

In summary, I believe this study provides valuable evidence supporting the prognostic relevance of discharge NIHSS. Further conceptual refinement and interdisciplinary integration may enhance its explanatory power and clinical impact in future research.

## Data Availability

Data sharing is not applicable to this article as no data were created or analysed in this study.
